# Supplementary material for: A Sub-Clone of RAW264.7-Cells Form Osteoclast-Like Cells Capable of Bone Resorption Faster than Parental RAW264.7 through Increased De Novo Expression and Nuclear Translocation of NFATc1
Source: Int J Mol Sci. 2020 Jan 14;21(2):538. doi: 10.3390/ijms21020538 (PMC7013577; doi:10.3390/ijms21020538)
Supplement: Supplementary file 1 [file ijms-21-00538-s001.pdf]

A sub-clone of RAW264.7-cells form osteoclast-like cells capable of bone resorption faster than parental RAW264.7 through increased *de novo* expression and nuclear translocation of NFATc1

Laia Mira-Pascual [1], Anh N. Tran [1,2], Göran Andersson [1], Tuomas Näreoja [1,*] and Pernilla Lång [1,*]

Supplementary material

Quantitation of large TRAP-positive cells (Figure 4) was done with an ImageJ-macro.

```
Stack.setDisplayMode("grayscale");
run("Z Project...", "start=2 projection=[Sum Slices]");
rename("SUM_ActiveR+G.jp2");
selectWindow("SUM_ActiveR+G.jp2");
run("Median...", "radius=1");
run("8-bit");
setThreshold(0, 30);
run("Analyze Particles...", "size=100-Infinity show=Outlines display include");
String.copyResults();
```

%The 100 pixel area corresponds to area of 85 µm^2 and average size of a mononuclear cell is between 50-300 µm^2, threshold for large (multinuclear) cells was set for 300 µm^2

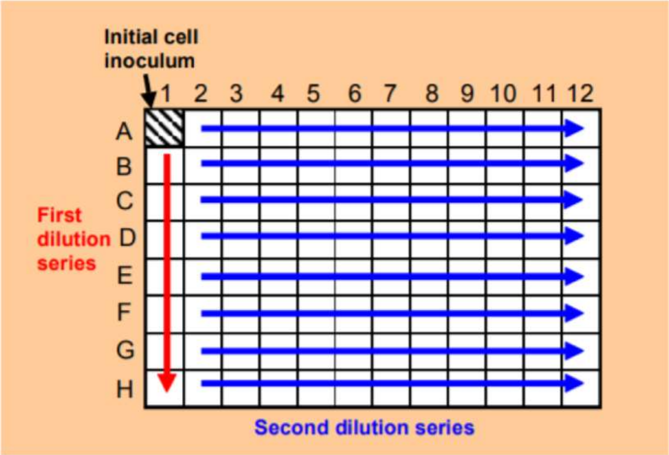

Figure S1. Dilution Scheme.

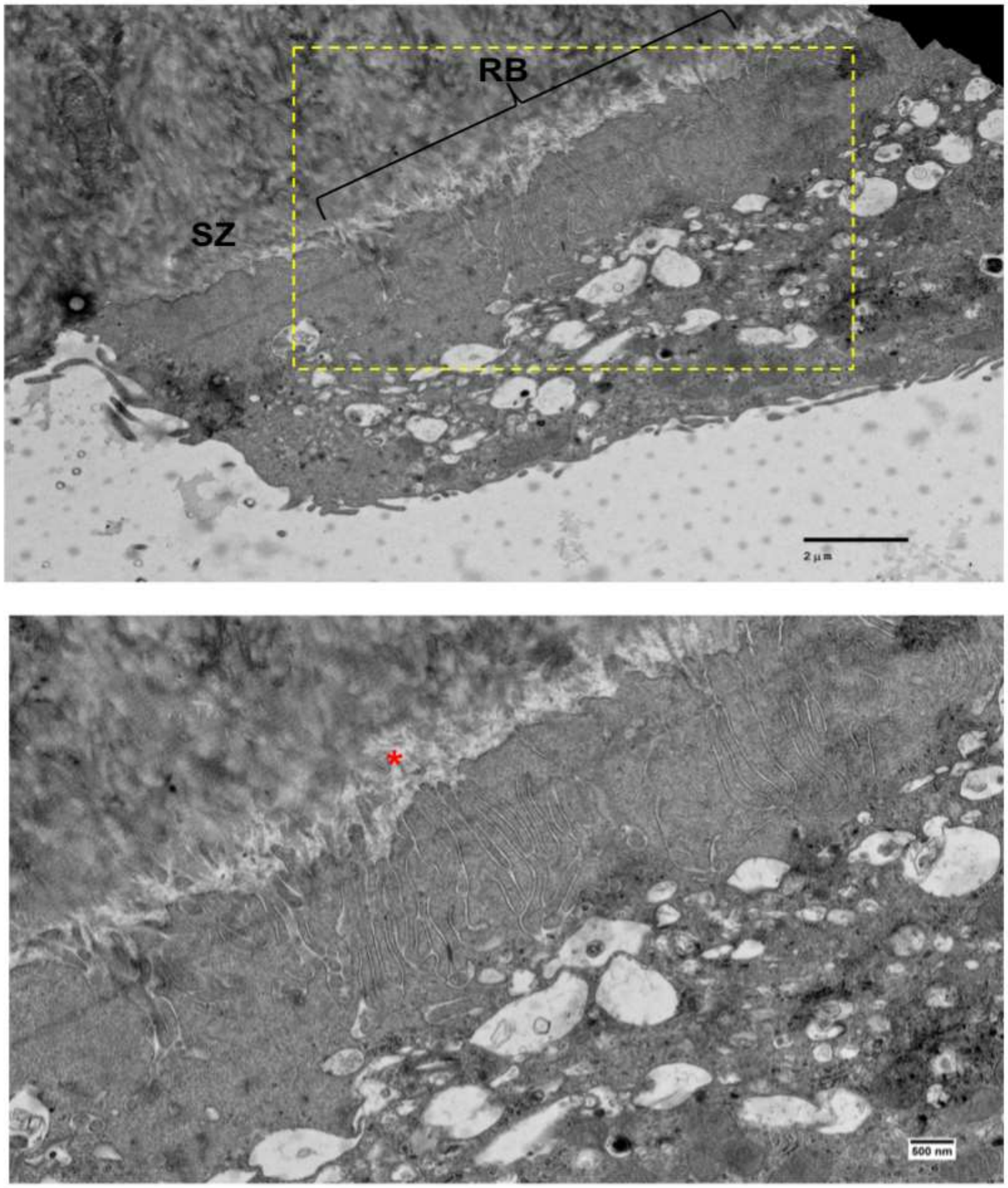

Figure S2. RAW264.7 macrophage cell line stimulated with RANKL forms RBs and SZs. Transmission electron micrograph of a RAW264.7 cell-derived osteoclast on dentine with a ruffled border (RB) and sealing zone (SZ). The bottom panel represents an enlarged image of the area outlined by the yellow dashed line in the top panel. Red asterisk shows some exposed collagen fibres, indicating some resorption has occurred. n=1.

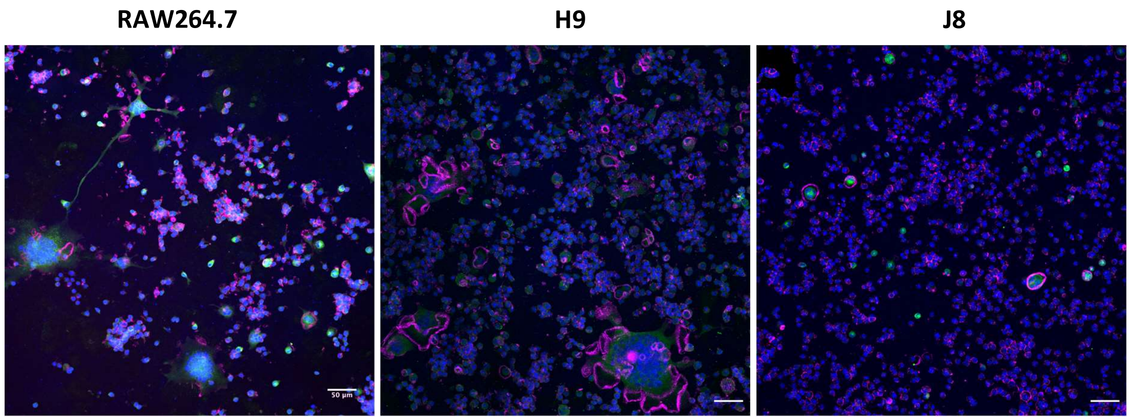

Figure S3. Sub-clone H9 forms more sealing zones than RAW264.7 or J8 on bone coating. Cells were cultured on bone coated coverslips for 10 days under 10 ng/ml RANKL stimulation and stained for nuclei (blue), TRAP (green) and f-actin (magenta). Thick circular f-actin bands were counted as sealing zones. Scale bar 50 µm.

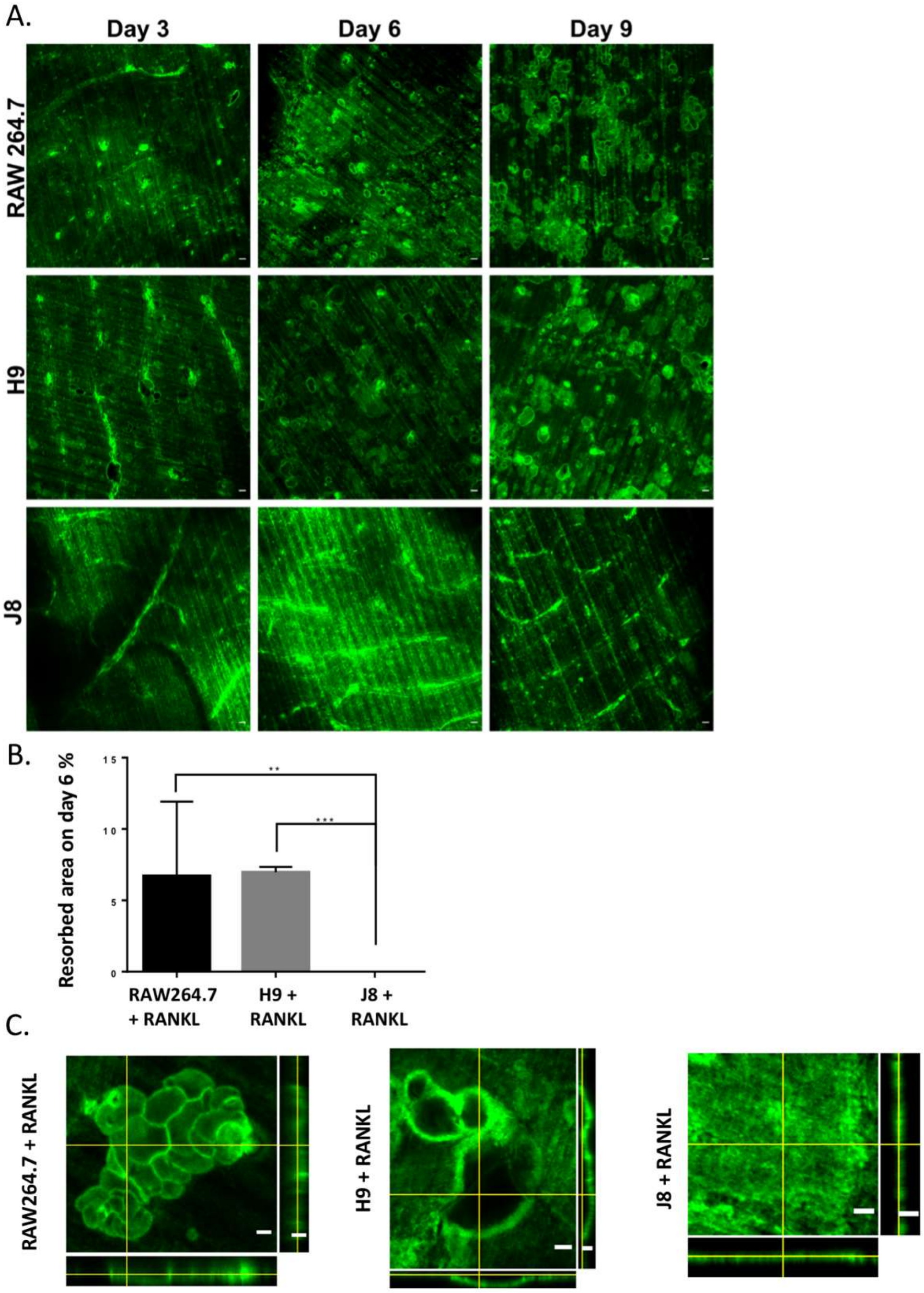

Figure S4. RAW264.7 and H9 form resorption pits on bovine bone slices. Bisphosphonate staining to identify resorbed areas on cortical bone (A), where RAW 264.7, H9, and J8 RANKL-stimulated cells were cultured for 3, 6, or 9 days. Scale bars, 10 µm. n=3. (B) Quantification of resorbed/acidified area on cortical bone day 6. (n=3) (C) Representative orthogonal views of the resorbed areas, indicating deeper pits in H9-cultures. Scale bar 10 µm both in x-y and x-z.

Table S1. Real time PCR primer pair information.

HTP-HM = high-throughput/heat mapping screening.

Primer	Sequence	Comment
TRAP (Forward)	GAC AAG AGG TTC CAG GAG ACC	
TRAP (Reverse)	GGG CTG GGG AAG TTC CAG	
Cathepsin K (Forward)	ACA GCA GGA TGT GGG TGT TCA	
Cathepsin K (Reverse)	GCC GAG AGA TTT CAT CCA CCT	
DC-stamp (Forward)	CGT GGG CCA GAA GTT GCT	
DC-stamp (Reverse)	GG CCA GTG CTG ACT AGG ATG A	
OC-stamp	NA	BioRad PrimerAssay
ATPv60d2 (Forward)	NA	BioRad PrimerAssay
ATPv60d2 (Reverse)	NA	BioRad PrimerAssay
Integrin b3	NA	BioRad PrimerAssay
ClC7 (Forward)	GCC TTC ATA GAG CCT GTT G	
ClC7 (Reverse)	CCT ACC ACA GAG AGA ATC AC	
M-CSF	NA	BioRad PrimerAssay
c-fms (Forward)	GCC CAG AAC TGG TTG TAG AGC	
c-fms (Reverse)	TTT CTT GTG GTC AGG GTG CTT C	
RANK (Forward)	TGG CTA CCA CTG GAA CTC AGA C	
RANK (Reverse)	TGC ACA CCG TAT CCT TGT TGA G	
NFATc1 (Forward)	TTG CTG CCC TTT CAC TGA TG	
NFATc1 (Reverse)	CCC TTT AAA AAT GAG GAC AAT AGC TTT	
Beta2microglubulin (Forward)	TAT GCT ATC CAG AAA ACC CCT CAA	Differentiation experiment reference gene
Beta2microglobulin (Reverse)	GCA GTT CAG TAT GTT CGG CTT C	Differentiation experiment reference gene
GAPDH (Forward)	TGA TGT CAT CAT ACT TGG CAG GTT	HTP-HM reference gene<br>Differentiation experiment reference gene
GAPDH (Reverse)	AAG GCT GTG GGC AAG GTC AT	HTP-HM reference gene<br>Differentiation experiment reference gene

Glucuronidase, Beta (Forward)	CCT TTC GTA CCA GCC ACT ATC C	HTP-HM reference gene
Glucuronidase, Beta (Reverse)	CAC ATC ACA ACC GCA GGG TG	HTP-HM reference gene
